# Supplementary figures and images for: Polygenic risk scores for major depressive disorder and neuroticism as predictors of antidepressant response: Meta-analysis of three treatment cohorts
Source: PLoS One. 2018 Sep 21;13(9):e0203896. doi: 10.1371/journal.pone.0203896 (PMC6150505; doi:10.1371/journal.pone.0203896)

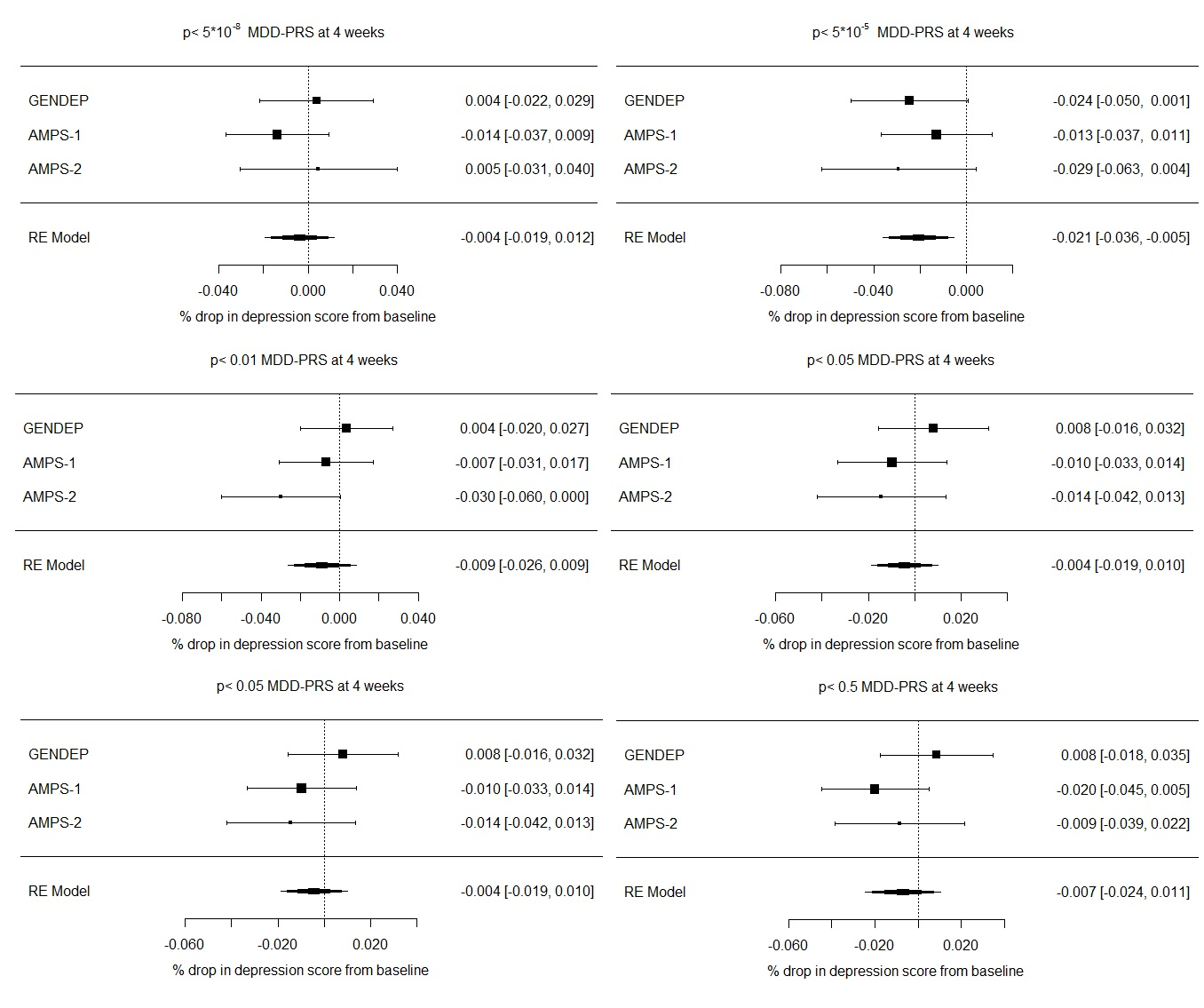

Supplement: S1 Fig — MDD PRS meta-analysis results at 4 weeks. A) p < 5*10−8, B) p < 5*10−5, C) p <0.01, D) p < 0.05, E) p < 0.1, F) p < 0.5. (TIF) [file pone.0203896.s006.tif]

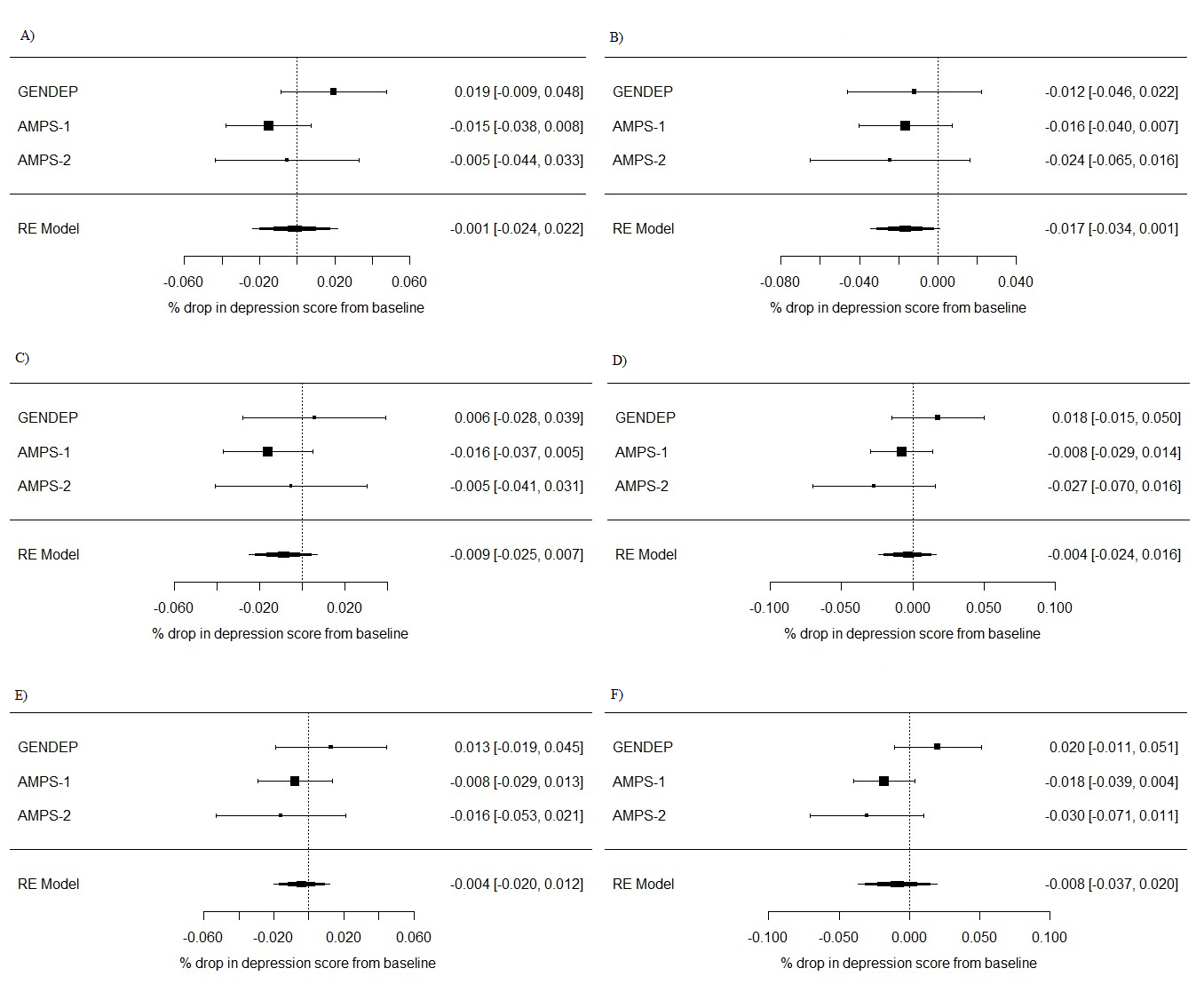

Supplement: S2 Fig — MDD PRS meta-analysis results at 8 weeks A) p < 5*10−8, B) p < 5*10−5, C) p <0.01, D) p < 0.05, E) p < 0.1, F) p < 0.5. (TIF) [file pone.0203896.s007.tif]

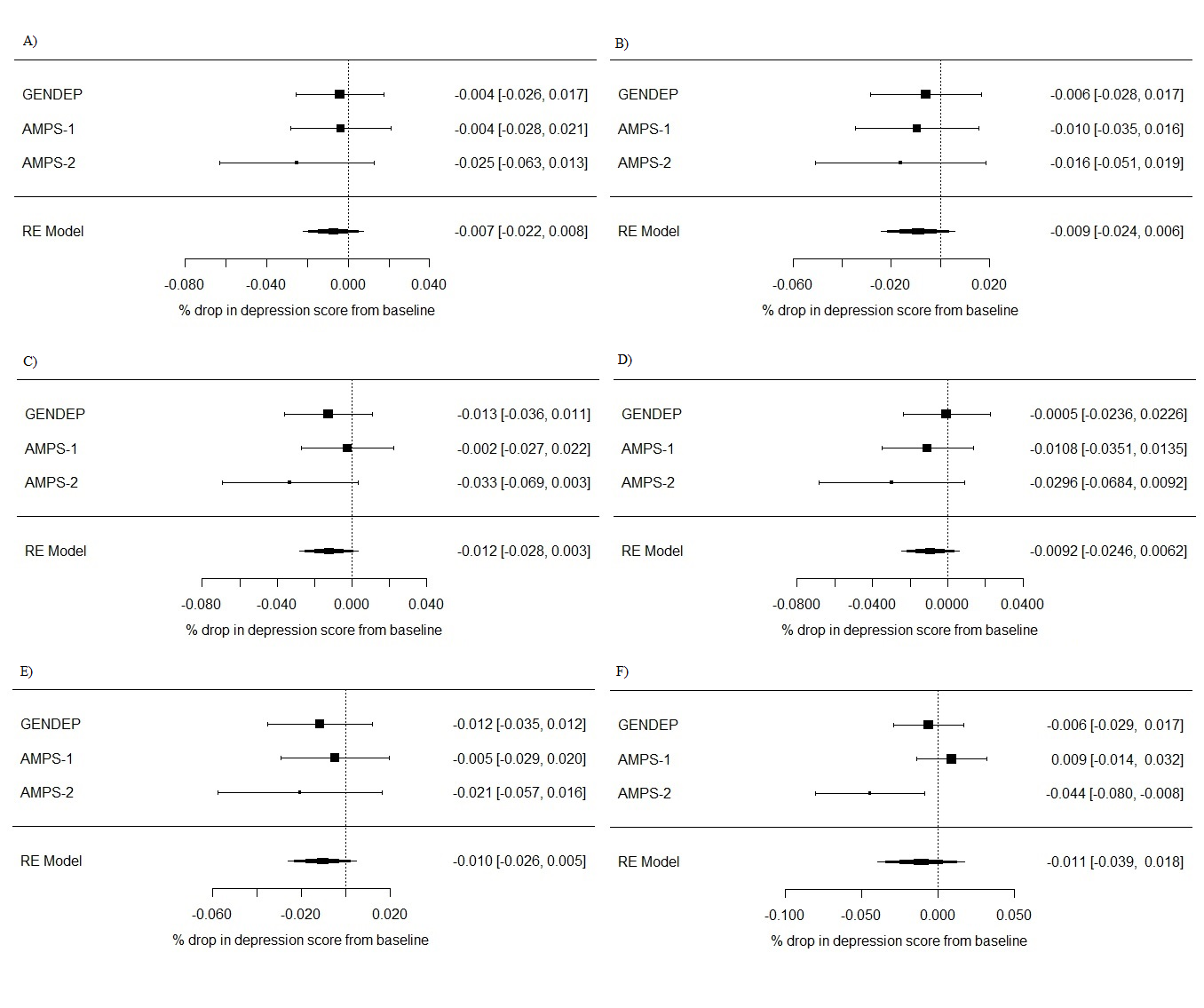

Supplement: S3 Fig — Neuroticism PRS meta-analysis results at 4 weeks A) p < 5*10−8, B) p < 5*10−5, C) p <0.01, D) p < 0.05, E) p < 0.1, F) p < 0.5. (TIF) [file pone.0203896.s008.tif]

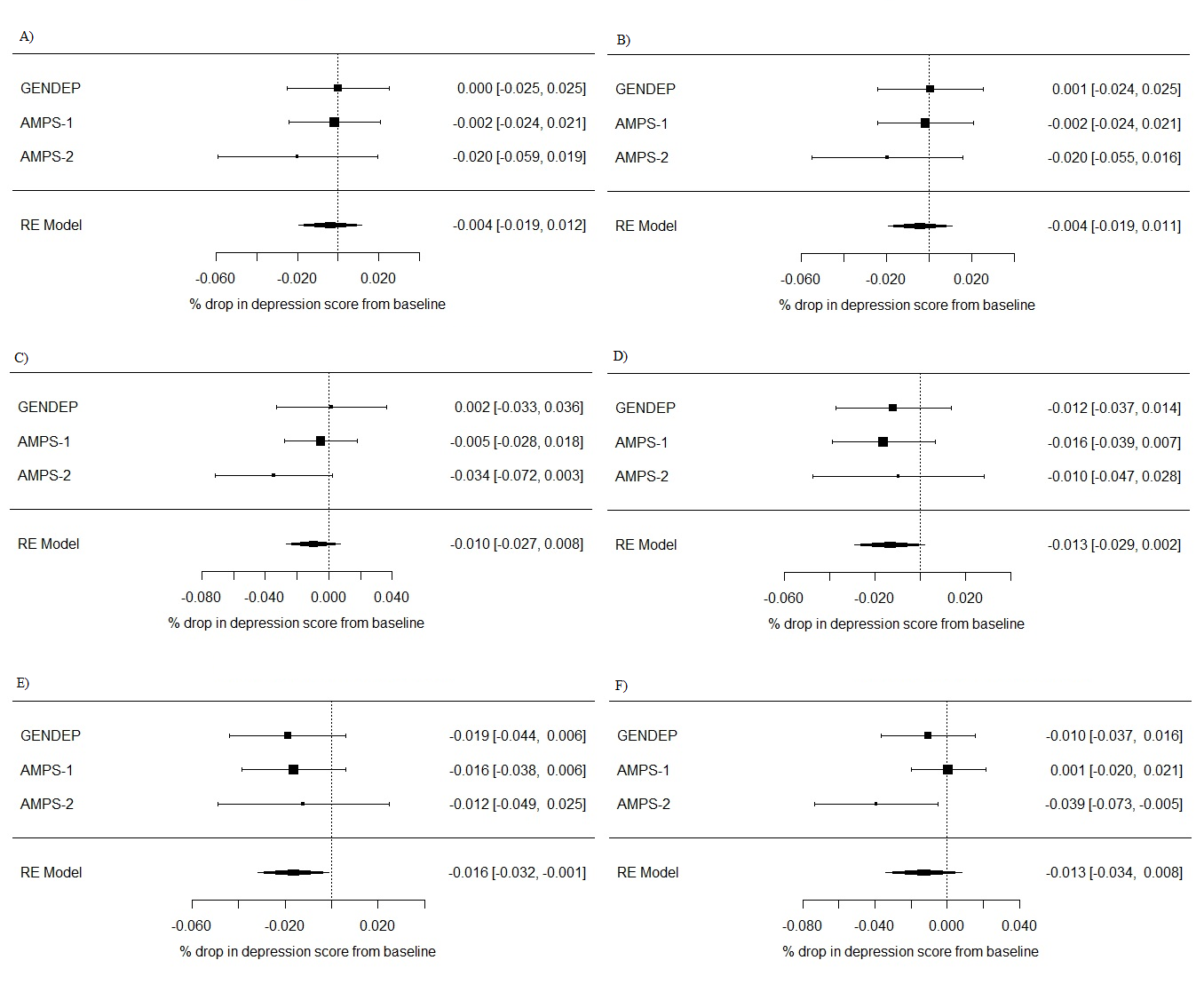

Supplement: S4 Fig — Neuroticism PRS meta-analysis results at 8 weeks A) p < 5*10−8, B) p < 5*10−5, C) p <0.01, D) p < 0.05, E) p < 0.1, F) p < 0.5.0. (TIF) [file pone.0203896.s009.tif]
